# Mono-ethylhexyl phthalate stimulates prostaglandin secretion in human placental macrophages and THP-1 cells

**DOI:** 10.1186/s12958-015-0046-8

**Published:** 2015-06-03

**Authors:** Lauren M Tetz, David M Aronoff, Rita Loch-Caruso

**Affiliations:** Environmental Health Sciences, University of Michigan, Ann Arbor, MI 48109 USA; Division of Infectious Diseases, Vanderbilt University Medical Center, Nashville, TN 37232 USA

**Keywords:** Placenta, Macrophage, Phthalates, Cyclooxygenase-2, Prostaglandin E2, Mono-ethylhexyl phthalate

## Abstract

**Background:**

Diethylhexyl phthalate (DEHP) is widely used as a plasticizer in polyvinyl chloride products. DEHP exposure, which is widespread in the US, increases preterm birth risk; however, the mechanisms driving this relationship are unclear. Because cyclooxygenase-2 (COX-2) dependent prostaglandin synthesis is implicated in preterm birth, we evaluated effects of mono-2-ethylhexyl phthalate (MEHP), the active metabolite of DEHP, on prostaglandin E2 (PGE2) synthesis and COX expression in human placental macrophages (PM). In addition, responses in PM were compared to those in a human macrophage-like cell line, THP-1.

**Methods:**

PM and THP-1 cells were treated for 2, 4, 8, or 24 h with MEHP concentrations ranging from 10 to 180 micromolar. PGE2 concentrations were assessed in culture medium using ELISA, and COX expression was determined by western blot.

**Results:**

Treatment of PM and THP-1 cells with 180 micromolar MEHP for 24 h significantly increased PGE2 release. Co-treatment of PMs or THP-1 cells with 180 micromolar MEHP and the non-selective COX inhibitor indomethacin reduced MEHP-stimulated PGE2 production. Similarly, co-treatment of PM and THP-1 cells with the COX-2 selective inhibitor NS-398 resulted in a significant decrease in PGE2, suggesting that MEHP-stimulated PGE2 is dependent specifically on increased COX-2 expression. Western blot analysis revealed a significant increase in COX-2 expression in PM and THP-1 cells treated with 180 micromolar MEHP, and no changes in COX-1 expression, supporting the role of COX-2 in MEHP-stimulated PGE2 synthesis.

**Conclusions:**

The findings from this study are the first to demonstrate phthalate-stimulated PGE2 synthesis in PM and warrant future studies into COX-2-dependent prostaglandin synthesis as a mechanism of toxicant-associated preterm birth.

**Electronic supplementary material:**

The online version of this article (doi:10.1186/s12958-015-0046-8) contains supplementary material, which is available to authorized users.

## Background

Preterm birth is a serious health problem, affecting 21 million infants worldwide and half a million per year in the US, and accounting for nearly one-third of all neonatal mortalities [1]. Intrauterine infection is the leading cause of preterm birth, but only represents 40–70 % of total preterm births [2, 3]. In fact, as many as half of preterm births are attributed to unknown causes [4]. A better understanding of contributing factors and mechanisms controlling untimely labor is necessary to prevent preterm birth and to improve maternal and fetal health.

A recent report from the Institute of Medicine highlights the importance of further research into factors contributing to preterm birth, including environmental factors such as pollutants [5]. Indeed, exposure during pregnancy to some environmental toxicants, including phthalate esters, increases risk for preterm birth [6–9].

Diethylhexyl phthalate (DEHP) is used widely as a plasticizer in polyvinyl chloride (PVC) consumer products. Because DEHP is not covalently bound to PVC, it is released into environmental media, such as water, food, or house dust. Following oral exposure, DEHP is quickly metabolized by gut lipases to its active metabolite, monoethylhexyl phthalate (MEHP), and excreted in the urine. A recent study conducted by the US Centers for Disease Control found measureable levels of MEHP in 80 % of urine samples analyzed, suggesting that exposure to DEHP is widespread in the U.S. population [10].

Previous studies show that women who are more highly exposed to DEHP during pregnancy have an increased risk for preterm birth and other adverse pregnancy outcomes including low birth weight and pregnancy loss [7, 11–14]. Measureable levels of DEHP metabolites, including MEHP, can be found in maternal urine and serum, placental tissue, fetal cord serum, and amniotic fluid, indicating that DEHP metabolites transfer from maternal blood to the fetal compartment [14–17]. Despite the mounting epidemiological evidence demonstrating an association between maternal DEHP exposure and increased risk for preterm birth, the mechanisms underlying these relationships are unclear.

Recent data collected by our laboratory suggest that MEHP stimulates expression in human trophoblast cells of *PTGS2,* the gene for COX-2 [18], an enzyme that is critical for synthesis of uterotonic prostaglandins, prostaglandin E_2_ (PGE_2_) and prostaglandin F_2α_ (PGF_2α_). COX-2 dependent prostaglandin synthesis is a critical event for the initiation of human parturition, regulating myometrial contractions and tissue remodeling in the gravid uterus [19]. Inhibition of prostaglandin synthesis following administration of COX-2 inhibitors delays parturition and prevents early labor in rodents, and *in vivo* exposure to bioactive prostaglandins induces myometrial contractions, cervical ripening and early labor, suggesting that untimely prostaglandin synthesis may drive preterm labor processes [19–22]. In humans, increases in amniotic fluid PGE_2_ and PGF_2α_ correspond with preterm labor and precede spontaneous labor at term [23, 24].

Macrophages within the uteroplacental environment are an important source of bioactive mediators including prostaglandins and cytokines. Placental and decidual macrophages express COX-2 and produce PGE_2_ in response to LPS or the pro-inflammatory cytokine IL-1β [25–29]. No studies to date have examined the effects of environmental toxicants, such as MEHP, on inducible COX-2 expression or prostaglandin secretion in macrophages from the utero-placental unit. However, several published studies suggest that MEHP influences immune function [30–32]. Therefore, in the current study, we test the hypothesis that MEHP increases prostaglandin secretion through induction of COX-2 expression in human primary placental macrophages (PMs) and in the human macrophage-like cell line, THP-1, to model primary placental and decidual macrophage behavior.

## Methods

This study was reviewed and approved by the Institutional Review Boards (IRBs) at the University of Michigan (#00035795, approval date 09/25/13) and Vanderbilt University (#131607, approval date 05/13/14). In compliance with the IRBs, the placental tissues collected for this study would otherwise have been discarded and the investigators did not collect any personal identifiable information or have direct interaction with subjects.

### Reagents

We purchased dimethyl sulfoxide (DMSO), indomethacin, and phorbol-12-myristate-13-acetate (PMA) from Sigma-Aldrich (St. Louis, MO, USA); charcoal-stripped fetal bovine serum (FBS) from HyClone Laboratories (Waltham, MA); RPMI 1640, Dulbecco’s Modified Eagle Medium (DMEM), penicillin/streptomycin solution, and phosphate buffered saline (PBS) from Life Technologies-Invitrogen (Carlsbad, CA); MEHP from Accustandard (New Haven, CT); LPS derived from *Salmonella typhimurium* from List Biological Laboratory (Campbell, CA); COX-1 and COX-2 monoclonal antibodies, and NS-398, from Cayman Chemical (Ann Arbor, MI); NONIDET P-40 Substitute from Research Products International Corp (Prospect, IL); and protease inhibitor tablets from Roche (Indianapolis, IN).

### Third trimester placental tissue acquisition

Placental tissue was collected from non-laboring women undergoing normal, medically-indicated cesarean section delivery between 37 and 39 weeks of gestation at the University of Michigan Women’s Hospital Birth Center or Vanderbilt University Medical Center. A total of 18 placentas were collected for placental macrophage isolation. Tissue samples collected at Vanderbilt University were provided by the Cooperative Human Tissue Network, which is funded by the National Cancer Institute. Exclusion criteria included the following: pre-eclampsia, diabetes, multi-fetal pregnancy, collagen vascular disease, cervical cerclage, immune-compromised conditions, bacterial vaginosis or clinical chorioamnionitis (as noted in the chart or suspected by attending physician), prescription of antibiotics in the past two weeks (with the exception of routine, pre-operative antibiotics), cigarette smoking, third trimester bleeding, major maternal medical conditions (e.g., chronic renal disease, sarcoidosis, hepatitis, HIV), or any condition requiring the tissues to undergo pathological examination.

### Placental macrophage isolation and culture

For placental macrophage isolations, a 30–60 g sample of tissue was excised from the placenta and then transferred to the laboratory in sterile PBS. Isolation of macrophages was performed as described previously [33]. Briefly, tissue was washed three times in PBS, digested to a single cell suspension and loaded on a 25 %/50 % Percoll gradient to remove cellular debris. Macrophages were isolated from single cell suspensions using MACS Miltenyi CD14 microbeads. Purity of freshly isolated macrophages was ~85 % on average, as assessed by percentage of viable, CD68+ cells, determined by flow cytometry (see Additional file 1: Figure S1, for flow cytometry data). After 24 h of adherence purification, purity of macrophage cultures was 95 % or above as determined by visual morphological inspection (see Additional file 1: Figure S1 for placental macrophage image). Yields of placental macrophage cells per gram weight of tissue ranged from 2e5–1e6 cells per gram weight of tissue. Cells were seeded at a density of 200,000 cells per well in a polystyrene, 24-well culture plate in RPMI with 1 % antibiotic/antimycotic solution and 10 % charcoal dextran FBS (RPMI +/+), and then incubated for 24 h in a humidified atmosphere at 37 °C and 5 % CO_2_. Before treatment, cells were visually inspected to verify proper morphology.

### THP-1 cell culture

We used the THP-1 cell line as a model to compare to primary placental macrophages because these cells are easily obtained, are genetically clonal, and represent a potential alternative model to primary cells for macrophage toxicity testing in cases where human placental tissues are difficult to obtain. The human monocytic leukemia cell line THP-1 was obtained from the American Type Culture Collection (ATCC, TIB-202; Manassas, VA, USA) and cultured as described previously [34]. THP-1 cells were used in this study to model placental and decidual macrophage behavior, as we have demonstrated previously [35]. THP-1 cells were seeded at a density of 200,000 cells per well in a 24-well plate, cultured overnight, and then differentiated into adherent, macrophage-like cells by culturing with 100 nM PMA in RPMI +/+ for 24 h at 37 °C and 5 % CO_2_. After differentiation and before treatment, cells were washed once with culture medium to remove residual PMA. PMA-treated THP-1 cells were used for all THP-1 cell experiments described in this study. For the indomethacin studies, THP-1 cells were seeded at a density of 200,000 cells per well in a 96-well plate and cultured for 24 h following PMA treatment in RPMI +/+, before co-treating with indomethacin and MEHP.

### Placental macrophage and THP-1 cell treatment

Primary PMs and THP-1 cells were treated with medium alone, 0.05 % DMSO (solvent control), or 10, 45, 90 or 180 μM MEHP in triplicate in RPMI 1640 medium (with L-glutamine without phenol red, supplemented with 100 U/mL penicillin and 100 μg/mL streptomycin) for 2, 4, 8, or 24 h in a humidified atmosphere at 37 °C and 5 % CO_2_. For PM experiments, experiments were repeated a minimum of 3 times, each time using cells isolated from one woman’s placental tissue, with treatments balanced across subjects. For THP-1 experiments, experiments were repeated a minimum of three times on different days. For indomethacin inhibitor experiments, cells were treated with solvent control or 180 μM MEHP and co-treated with either 1 or 10 μM indomethacin, a non-selective COX inhibitor, for 24 h. For COX-2 selective inhibition experiments, cells were pretreated with 1 μM NS-398 for 30 min, and then treated for 24 h with solvent control or 180 μM MEHP with or without 1 μM NS-398. Effective inhibitor concentrations were identified based on preliminary concentration response curves in THP-1 cells, using 500 ng/mL LPS as an inducer of COX-2 (data not shown). Concentrations of MEHP used for the current study were sub-cytotoxic, as determined by a lactate dehydrogenase assay on treatment medium from PM and THP-1 cells treated with 0, 45, 90, and 180 μM MEHP for 24 h (data not shown).

### Prostaglandin quantification

We measured MEHP-stimulated prostaglandin secretion in human primary PM cells and THP-1 cells in culture. PGE_2_ and PGF_2α_ concentrations in the culture medium were quantified with enzyme immunoassay (EIA) following the manufacturer’s recommended protocol (Cayman Chemical, Ann Arbor, MI, USA).

### Western immunoblot for COX-1 and COX-2 protein expression

THP-1 cells were seeded at a density of 5e6 cells per dish in a 60-mm polystyrene culture dish and PMs were seeded at a density of 10e6 cells per well in a 6-well polystyrene culture plate. Following 24 h incubation, cells were treated for 8 h with solvent control (0.05 % DMSO), 180 μM MEHP, or 500 ng/mL LPS. Following treatment, cells were lysed with ice-cold lysis buffer (0.5 % NONIDET P-40 Substitute, 250 mM NaCl, 50 mM Tris–HCl, with 2X protease inhibitor tablets). Protein was separated on a 10 % polyacrylamide gel and then transferred to a PVDF membrane. All samples were run alongside COX-1 and COX-2 positive control lysates (human COX-2 control lysates were obtained from Cayman chemical and COX-1 control lysates were a gift from Dr. William L. Smith, University of Michigan). Membranes were probed for COX-1 (anti-human COX-1 mouse monoclonal antibody; 5 μg/mL), COX-2 (anti-human COX-2 mouse monoclonal antibody; 0.5 μg/mL) and α-tubulin (anti-α-tubulin mouse monoclonal antibody; 1:40,000) or GAPDH (anti-GAPDH rabbit monoclonal antibody; 1:1000).

### Statistical analysis

Data for each cytokine were expressed as pg/ml and analyzed by ANOVA to test for treatment effect, followed by Tukey *post hoc* pairwise comparison of means when ANOVAs were statistically significant. For experiments with replicates within an experimental day, mixed model ANOVA was used with treatment as the fixed variable and experiment day as the random variable. Data are expressed as the mean ± SE of 3 different experimental days for THP-1 cells or 3–4 women, depending on the experiment, for placental macrophages.

## Results

### MEHP-stimulated prostaglandin synthesis in placental macrophage cultures

**Fig. 1:**
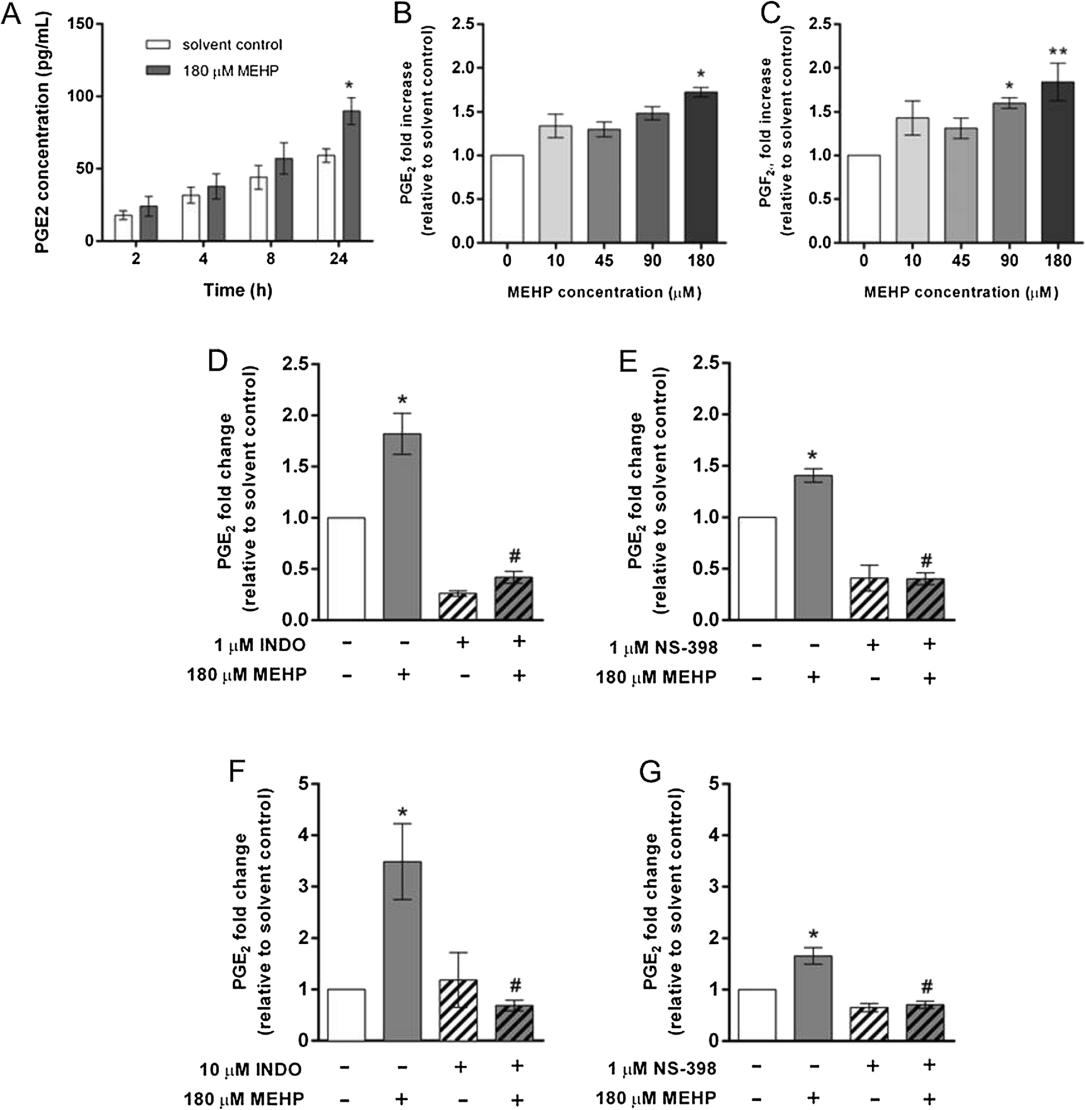
Effects of MEHP with and without indomethacin (INDO) or NS-398 on prostaglandin release from primary placental macrophage cells or THP-1 cells. **a** Placental macrophages were treated for 2, 4, 8, or 24 h with DMSO (0.05 % v/v; solvent control) or 180 μM MEHP. **b** and **c** Placental macrophages were treated for 24 h with 0 (0.05 % v/v DMSO; solvent control), 10, 45, 90, or 180 μM MEHP. **d** and **e** Placental macrophages were treated for 24 h with DMSO (0.05 % v/v; solvent control) or 180 μM MEHP with or without 1 μM indomethacin or 1 μM NS-398. **f** and **g** THP-1 cells were treated for 24 h with DMSO (0.05 % v/v; solvent control) or 180 μM MEHP with or without 10 μM indomethacin or 1 μM NS-398. EIAs for PGE_2_ or PGF_2α_ were performed on cell culture medium as described in the “Methods” section. N = 3-4 women per placental macrophage experiment, 1–3 replicates within each treatment group per experiment. N = 3 experiments for THP-1 cells. *p < 0.05, **p < 0.01, compared to solvent controls

Treatment of primary PMs with 180 μM MEHP but not 10, 45, or 90 μM MEHP for 24 h significantly increased PGE_2_ release by 1.7-fold (Fig. 1a,b). Furthermore, treatment with 90 or 180 μM MEHP significantly stimulated PGF_2α_ release from PM cells by approximately 1.5 and 1.8-fold, respectively (Fig. 1c). We observed no statistically significant effect of 180 μM MEHP on PGE_2_ concentrations after 2, 4, or 8 h compared to solvent controls (Fig. 1a). However, there was a trend suggestive of increased PGE_2_ at all time-points measured. The effects of 180 μM MEHP treatment on PGE_2_ synthesis were reproducible in THP-1 cells used to model PM behavior (Fig. 1f,g).

### Effects of COX inhibitors on MEHP-stimulated PGE_2_ release

Co-treatment with 1 μM indomethacin, a non-selective COX inhibitor, significantly decreased MEHP-stimulated PGE_2_ release in PMs and THP-1 cells by 77 % and 60 %, respectively, compared to MEHP alone, confirming COX-dependent PGE_2_ synthesis with MEHP stimulation (Fig. 1d,f). Furthermore, co-treatment of PMs or THP-1 cells with the COX-2 selective inhibitor NS-398 resulted in significant decreases in PGE_2_ concentrations of 70 % and 57 %, respectively, compared with MEHP alone, suggesting that MEHP-stimulated PGE_2_ synthesis is dependent on COX-2 activity, specifically.

### MEHP effects on COX-1 and COX-2 protein expression in placental macrophage and THP-1 cells

**Fig. 2:**
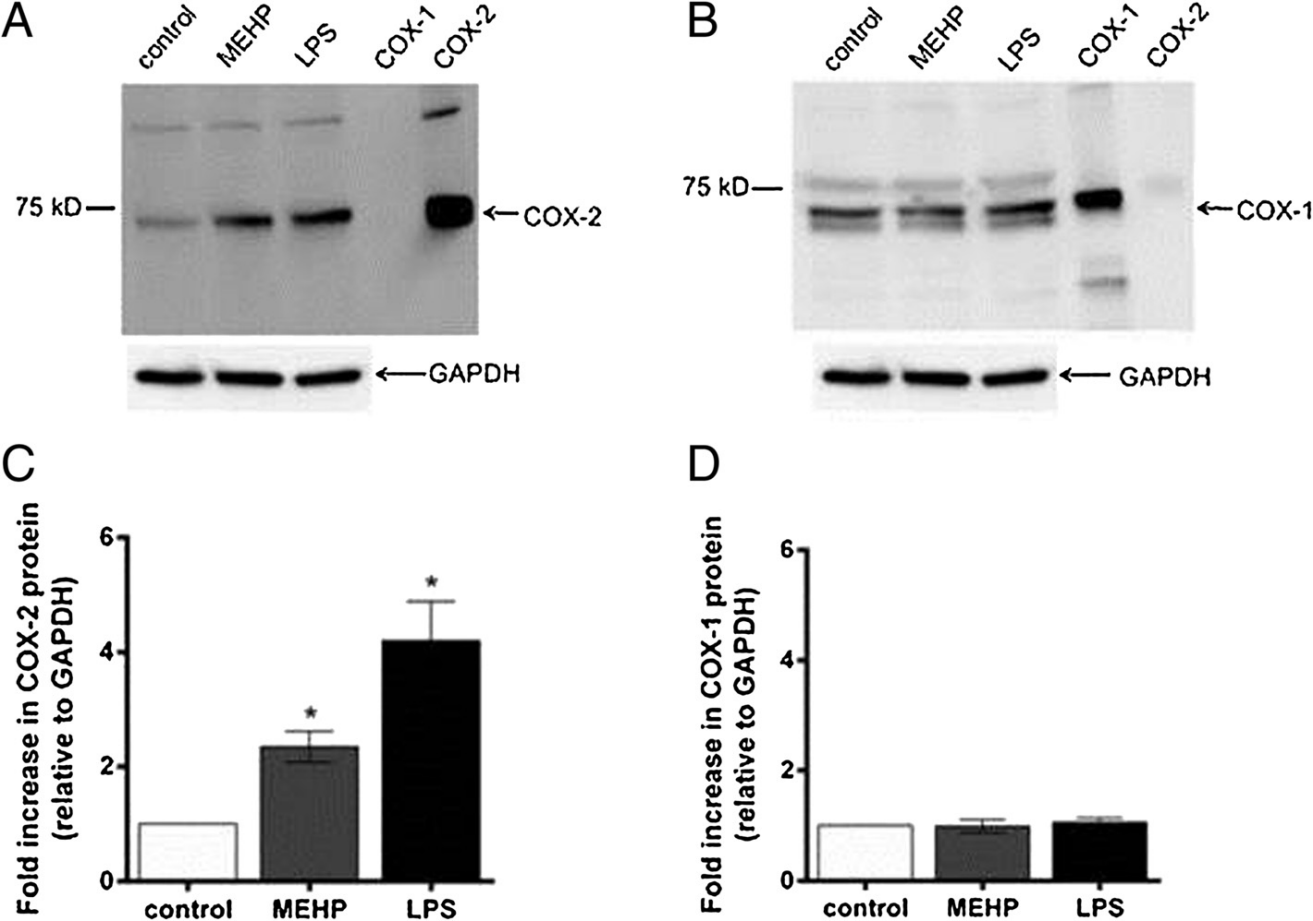
MEHP effects on COX-1 and COX-2 protein expression in placental macrophages. Western blot and densitometry analysis of COX-2 (**a** and **c**) and COX-1 (**b** and **d**) in placental macrophages treated for 8 h with DMSO (0.05 % v/v; solvent control), 180 μM MEHP or 500 ng/mL LPS. Western blot and densitometry analysis was performed on placental macrophage lysates as described in the “Methods” section. All samples were run alongside COX-1 and COX-2 positive control lysates, seen on the far right of the gel. N = 3 women. *p < 0.05, compared to solvent controls

**Fig. 3:**
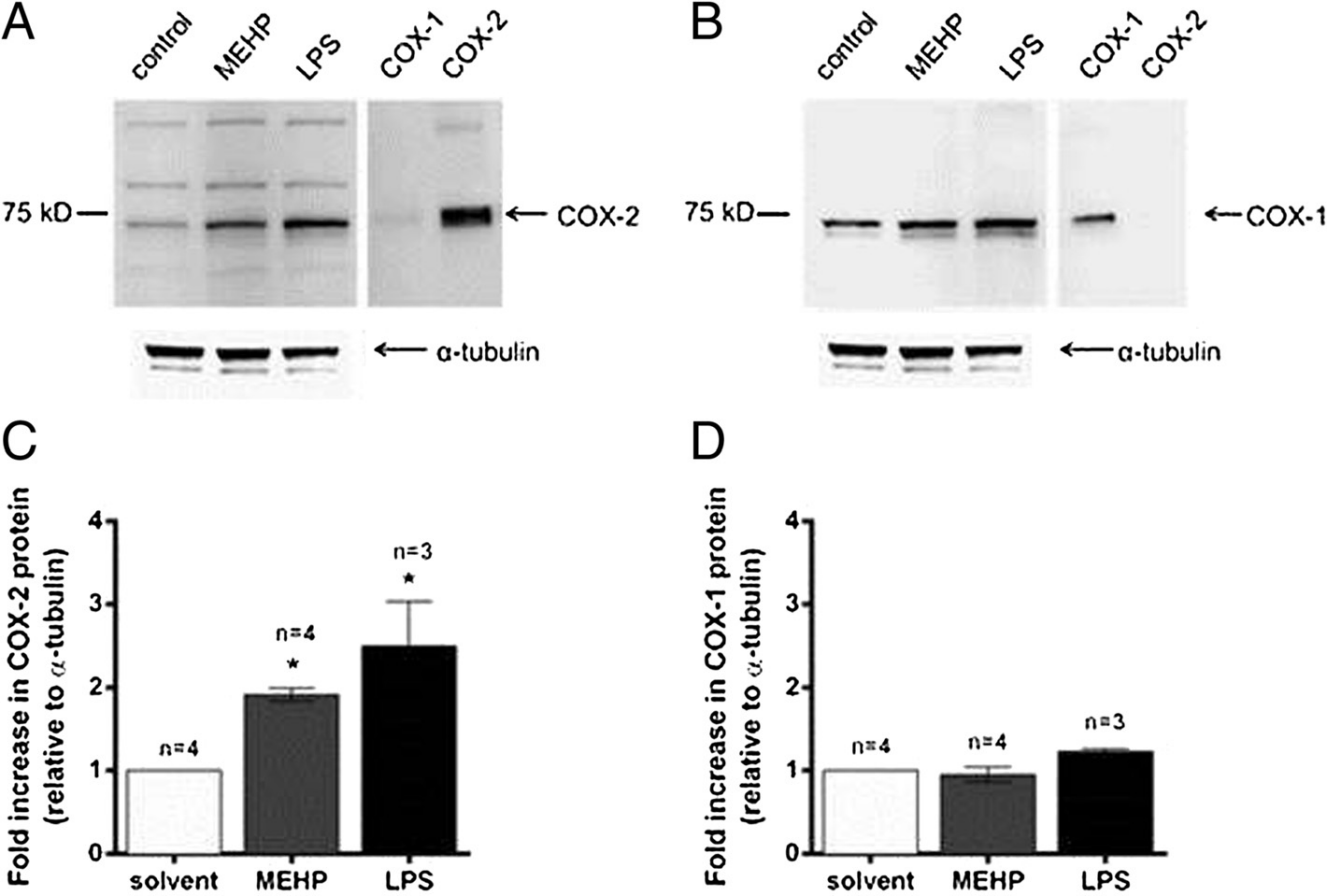
MEHP effects on COX-1 and COX-2 protein expression in THP-1 cells. Western blot and densitometry analysis of COX-2 (**a** and **c**) and COX-1 (**b** and **d**) in THP-1 cells treated for 8 h with DMSO (0.05 % v/v; solvent control), 180 μM MEHP or 500 ng/mL LPS. Western blot analysis was performed on THP-1 cell lysates as described in the “Methods” section. All samples were run alongside COX-1 and COX-2 positive control lysates, seen on the far right of the gel. *N* = 3–4 experiments. *p < 0.05, compared to solvent controls

Western blot densitometric analysis revealed a significant increase in COX-2 protein expression of approximately 2-fold in PMs treated with 180 μM MEHP (Fig. 2a,c) but no significant changes in COX-1 expression (Fig. 2b,c), supporting the role of COX-2 in MEHP-stimulated PGE_2_ synthesis. MEHP induction of COX-2 was also observed in THP-1 cells (Fig. 3). Furthermore, 180 μM MEHP induced COX-2 expression at levels comparable to those observed with a potent pro-inflammatory stimulus, lipopolysaccharide (LPS; Figs. 2 and 3).

## Discussion

Exposure during pregnancy to the common environmental contaminant DEHP has been associated in epidemiologic studies with increased risk for preterm birth [7, 11]. Because immune cells can be important sources of uterotonic prostaglandins [36], the objective of the current study was to determine the influence of MEHP, a principle bioactive metabolite of DEHP, on prostaglandin synthesis in primary human PMs. Our results demonstrate that MEHP-stimulated synthesis of uterotonic prostaglandins is dependent on the activity of inducible COX-2 enzyme in both PMs and THP-1 cells. Furthermore, MEHP induced COX-2 expression at levels comparable to those observed with a potent pro-inflammatory stimulus, lipopolysaccharide. We observed significant effects with MEHP concentrations ranging from 90 to 180 μM. These concentrations are within one order of magnitude of average MEHP concentrations reported by Lin et al. [37] in human umbilical cord serum (35.7 μM) and human maternal serum (42.6 μM), but are higher than concentrations reported by Latini et al. [11] in their study of MEHP in umbilical cord serum (average of 1.8 ± 2.2 μM, range of 0–10.6 μM). To our knowledge, this study represents the first to examine effects of a reproductive toxicant on PMs. These findings support MEHP-stimulated prostaglandin synthesis and induction of COX-2 enzyme in PMs as a potential mechanism for MEHP-associated preterm birth. Moreover, the similar responses we observed with the human monocyte cell line THP-1 suggest that MEHP may have a general immunomodulatory effect on prostaglandin synthesis, which may have implications for other disease outcomes as well.

The findings from the present study are concordant with our previous findings demonstrating an increase in *PTGS2* mRNA expression in placental trophoblast cells with 90 and 180 μM MEHP [18]. In addition, MEHP exposure has previously been shown to induce expression of COX-2 in immortalized murine liver cells, murine spermatocytes, and rat alveolar macrophages at concentrations ranging from 200 to 1000 μM MEHP [38–40]. Notably, the present study includes the lowest effective concentration yet reported for MEHP effects on COX-2 expression in an immune cell, to the best of our knowledge.

Contrary to the findings from the present study, Xu et al. observed decreased COX-2 expression in the junctional zone of placenta from pregnant rats dosed with 750 or 1500 mg/kg DEHP daily from gestational day 0 to 19 [41]. The discrepancy between the current study and the findings of Xu et al. may be explained by differences in *in vitro* culture conditions and *in vivo* exposure, differences in responses to MEHP between species, differences in cellular response due to chronic *in vivo*, vs. acute *in vitro* exposure, or differences in MEHP responses of whole placental tissue compared to isolated placental macrophage cells. Furthermore, although the lowest effective dose of DEHP given in the Xu et al. study resulted in a serum MEHP concentration of 234 μM, similar to the highest concentration used in the current study, a significant amount of DEHP remained in the rat serum following exposure. Therefore, the observed differences could also be due to the additional presence of DEHP in the serum of exposed rats.

The investigations presented here utilized both primary placental macrophages and the human macrophage-like THP-1 cell line. As noted in previous studies, THP-1 cells respond similarly compared to primary placental and decidual macrophages in their response to bacterial infection [35]. However, no studies to date have compared THP-1 cells to primary placental macrophages in their ability to respond to reproductive toxicants. Our data are the first to demonstrate that THP-1 cells respond similarly to primary placental macrophages in their secretion of prostaglandins and induction of COX expression in response to MEHP treatment. Thus, THP-1 cells may represent a facile model of placental macrophages for investigating the influence of environmental toxicants on macrophage function.

The mechanism of MEHP-stimulated prostaglandin synthesis in placental cells remains to be elucidated; however, recent evidence suggests that PPAR may be important for MEHP modulation of inflammatory responses [31, 32]. DEHP and MEHP are putative PPAR activators, and several studies show that MEHP-mediated toxicity is dependent on PPAR in a variety of tissue and cell types [31, 42]. Furthermore, because PPAR is an important regulator of inflammatory responses in macrophages, future studies could examine PPAR activation as a potential mechanism for MEHP-stimulated prostaglandin synthesis in primary PMs [43].

In addition to effects on labor processes, MEHP-stimulation of PGE_2_ synthesis may have implications for innate immune function in the female reproductive tract. Previous findings from our group suggest that exaggerated levels of PGE_2_ suppress immune responses against intrauterine Group A *Streptococcus* infection [35]_._ Because intrauterine infection is an important causal factor for preterm labor, future studies could examine the influence of MEHP-stimulated PGE_2_ synthesis on the progression of intrauterine infections.

## Conclusions

The findings from the current study demonstrate COX-2-dependent stimulation of PGE_2_ by MEHP in primary human PMs and THP-1 cells, suggesting that PMs may be an important target of MEHP toxicity. Because COX-2 dependent prostaglandin synthesis is implicated in preterm labor processes, these findings may represent a potential mechanism by which MEHP contributes to risk for preterm birth. Furthermore, these findings support future epidemiological research examining the influence of DEHP exposure on prostaglandin synthesis in the uteroplacental unit.

## Additional file

Additional file 1: Figure S1. Placental Macrophage Culture Purity. In this file, we have included the flow cytometry data from freshly isolated macrophages showing CD68^+^ cell percentages and an image of the placental macrophage cultures after 24 h of adherence purification.
