# Supplementary material for: Mono-ethylhexyl phthalate stimulates prostaglandin secretion in human placental macrophages and THP-1 cells
Source: Reprod Biol Endocrinol. 2015 Jun 3;13:56. doi: 10.1186/s12958-015-0046-8 (PMC4462084; doi:10.1186/s12958-015-0046-8)
Supplement: Additional file 1: Figure S1. — Placental Macrophage Culture Purity. In this file, we have included the flow cytometry data from freshly isolated macrophages showing CD68+ cell percentages and an image of the placental macrophage cultures after 24 h of adherence purification. [file 12958_2015_46_MOESM1_ESM.zip › 12958_2015_46_add1.rtf]

Additional File 1, Supplemental Figure S1. Placental macrophage culture purity. Flow cytometric analysis of freshly isolated placental macrophages from two separate subjects (A and B). Cells were stained with Aqua dead cell stain and PE-conjugated CD68 antibody and analyzed by flow cytometry as described in “Methods”. C) Representative image of placental macrophages after adherence purification (24 hours of culture) (Image magnified from original 10x image).
Additional Files, Methods

Flow cytometry 
Following placental macrophage isolation, cells were distributed to flow cytometry tubes, 1 million cells per tube. Cells were stained for 30 min at 4°C with LIVE/DEAD fixable aqua dead cell stain (Invitrogen). Following dead cell staining, cells were washed with PBS containing 1% BSA (FACS buffer), then fixed and permeabilized in fixation/permeabilization solution (BD Biosciences) for 15 min at 4°C and washed in permeabilization wash buffer (BD Biosciences). Cells were then stained for 15 minutes at 4°C with anti-human pan-macrophage marker, CD68 (eBiosciences). Isotype controls were included in separate tubes.  Following staining, cells were washed and resuspended in FACS buffer and immediately acquired on a BD LSR II (BD Biosciences). Analysis was performed with BD FACSDiva software (BD Biosciences). Total cells were gated first on viability as indicated by cells negative for Aqua stain, and then further gated for CD68 positive cells.
